# Spermine enhances the activity of anti-tuberculosis drugs

**DOI:** 10.1128/spectrum.03568-23

**Published:** 2023-12-14

**Authors:** Carine Sao Emani, Norbert Reiling

**Affiliations:** 1 Microbial Interface Biology, Research Center Borstel, Leibniz Lung Center, Borstel, Germany; 2 German Center for Infection Research (DZIF), Partner Site Hamburg-Lübeck-Borstel-Riems, Borstel, Germany; CNRS - University of Toulouse, Toulouse, France

**Keywords:** tuberculosis (TB), *Mycobacterium tuberculosis *(M.tb), spermine (Spm), reactive oxygen species (ROS), cumene hydroperoxide (CuOOH), isoniazid (INH), rifampicin (RIF), para-aminosalicylic acid (PAS), bedaquiline (BDQ)

## Abstract

The discovery of molecules that could shorten the treatment period of tuberculosis (TB) holds the promise of improving the current regimen. Polyamines are organic polycationic alkylamines found in millimolar concentrations in all living cells. Spermine (Spm) is a polyamine that was shown (more than 70 years ago) to be toxic to *Mycobacterium tuberculosis* (M.tb). In this study, we used various biochemical assays to assess the mechanism of action of Spm. Furthermore, using various drug susceptibility testing methods, we investigated the ability of Spm to enhance the activity of first-line TB drugs, isoniazid (INH) and rifampicin (RIF), as well as second-line TB drugs, para-aminosalicylic acid (PAS) and bedaquiline (BDQ). Our findings revealed that at toxic concentrations, Spm induced the formation of reactive oxygen species (ROS), a reaction that was influenced by the pH and the iron concentration of the media. Furthermore, the generation of ROS by Spm was associated with increased ferric iron and NADP levels. Moreover, the ROS produced by Spm synergized with the ROS generated by the lipophilic peroxide cumene hydroperoxide (CuOOH), as well as with the ROS generated by RIF and PAS, while enhancing their antimycobacterial activity. Additionally, we found that Spm enhanced the combined activity of INH and RIF against M.tb. Lastly, Spm was also able to enhance the activity of BDQ due to another mechanism of action. This study provides data and findings that would fuel future studies on the suitability of Spm as a potential adjunct TB therapy. The enhanced antimycobacterial activity of TB drugs in combination with Spm could offer promising new avenues for improving TB treatment outcomes.

**IMPORTANCE:**

This is the first study that attempted to demonstrate the mechanisms of reactive oxygen species (ROS) generation by spermine (Spm) in *Mycobacterium tuberculosis* (M.tb). Furthermore, this is the first study to demonstrate that it is able to enhance the activity of currently available and World Health Organization (WHO)-approved tuberculosis (TB) drugs. Spermine can easily be obtained since it is already found in our diet. Moreover, as opposed to conventional antibiotics, it is less toxic to humans since it is found in millimolar concentrations in the body. Finally, with the difficulty of curing TB with conventional antibiotics, this study suggests that less toxic molecules, such as Spm, could in a long-term perspective be incorporated in a TB regimen to boost the treatment.

## INTRODUCTION

Tuberculosis (TB) was the leading cause of death caused by an infectious agent before the coronavirus (COVID-19) pandemic (1). Efforts to track the TB epidemic and reinforce the control and eradication of TB were hampered by the coronavirus pandemic (2). To date, an estimate of a quarter of the world population is infected with TB (3). About 85% of people can be cured with the current TB regimen (2). The current regimen of drug-susceptible TB takes an overall 6 months starting with a 2-month treatment with isoniazid (INH), rifampicin (RIF), ethambutol (ETB), and pyrazinamide (PZN), followed by a 4-month treatment with RIF and INH only. There are other regimens designed for the treatment of TB; however, the shortest regimen lasts at least 4 months, and all regimens include RIF and INH as first-line drugs (2). Irrespective of the regimen used for the treatment of drug-susceptible TB, the treatment is lengthy and requires more than two antibiotics. Besides the alteration in the microbial diversity of the gut and downstream consequences such as acquired susceptibility to *Clostridioides difficile* and others (4), they do have unpleasant side effects, increasing the probability of poor compliance by patients. This results in the adaptation of the surviving *Mycobacterium tuberculosis* (M.tb) sub-population in the patient by the acquisition of mutations that enable them to resist antibiotics, leading to drug-resistant TB (5 – 7). The treatment of drug-resistant TB [multidrug-resistant TB (MDR) and extensively drug-resistant TB (XDR)] consists of fluoroquinolones, second-line injectables, pretomanid, ethionamide, linezolid, and cycloserine, and the newly approved antibiotic bedaquiline (BDQ) (2, 8). Despite the recent discovery of a more promising regimen (9), the treatment of MDR and XDR TB is generally more cumbersome and longer. Though, BDQ has been recently approved, BDQ-resistant TB cases, have already been reported (10). One way to overcome this challenge is to genetically modify M.tb in order to investigate, identify, and validate new TB drug targets whose potential inhibitors can speed up and boost the current regimen (11 – 14). A second approach is to extract and investigate natural compounds from the environment (sea, soil, and plants) that could sometimes be even more potent than the current drugs (15, 16). A third approach is to investigate host enzymes that can be modulated during TB treatment to booster therapy (17 – 19). A final approach is to investigate the potential to enhance the current TB regimen with naturally occurring and readily available antimicrobial molecules that are less toxic than antibiotics (20, 21). Polyamines (PAs) are organic polycationic alkylamines found in millimolar concentrations in all living cells. Their main source is from the diet; however, they can be provided by the intestinal flora or produced through *de novo* synthesis in humans (22). There are three major polyamines, spermine (Spm), spermidine (Spd), and putrescine (Ptc). Because of their positively charged amino groups, they have a versatile biological role. They are able to alter cell signaling, DNA binding, transcription, RNA splicing, cytoskeleton functioning, electrochemical equilibrium regulation, electric excitation, and cardiac activity by modulating the activity of K^+^ channels and many other cellular processes (3). The antimycobacterial activity of Spm was first identified and investigated by James G. Hirsch and René J. Dubos in 1951 (23, 24). They initially showed that an extract of animal tissues had antimycobacterial activities. They further extracted and crystallized the molecule responsible for that activity and showed that it was Spm phosphate (23, 24). In later studies, they tested the activity of other amine compounds against mycobacteria and found that only Spm and Spd exerted antimycobacterial activities (23, 24). Since then, there have not been any follow-up studies on the potential antimycobacterial activity of Spm. We have recently shown that Spm is indeed active against M.tb and even better in culture media lacking albumin (25). However, it remains unclear how Spm exerts its activity against M.tb and, more so, if it is able to enhance the activity of known anti-TB drugs. Therefore, in this study, we attempted to elucidate the mechanism of action of Spm and demonstrated that it can enhance the activity of anti-tuberculosis drugs that are included in the World Health Organization (WHO)-approved TB regimen. This study provides a rationale to investigate if the targeted administration of Spm may represent a novel option for an adjunct TB therapy.

## MATERIALS AND METHODS

### Bacteria and culture conditions

*Mycobacterium tuberculosis* H37Rv used in this study was a laboratory stock ATCC (27294) made in the commercially available Sauton’s media (HiMedia Laboratories Pvt. Ltd. A-516) that was prepared with glycerol (0.02%) and tyloxapol (0.05%). All experiments in this study were performed in Sauton’s media [except one experiment that was performed in phosphate-buffered saline (PBS) for the purpose described below], due to previous studies indicating the conjugation of Spm with albumin (26), which is also found in the supplement of the conventional 7H9 media (manuscript under revision). Mycobacteria (pCherry10) (27, 28) expressing (under P_smyc_) mCherry [a variant of the *Discosoma* sp. red fluorescent protein (D*s*Red)] (29) were used as well in this study. For colony-forming units (CFUs) enumeration, bacteria were plated on Middlebrook 7H11 Agar Base media (Sigma-Aldrich Chemie GmbH) supplemented with either the laboratory-made ADS supplement (0.005 g/mL bovine albumin fraction V, 0.002 g/mL dextrose, and 0.85 mg/mL sodium chloride) or the commercially ready-made OADC supplement (0.05 mg/mL oleic acid, 0.005 g/mL bovine albumin fraction V, 0.002 g/mL dextrose, 0.004 mg/mL catalase, and 0.85 mg/mL sodium chloride). Dihydro-dichlorofluorescein (H2DCF), cumene hydroperoxide (CuOOH), tert-butylnitrite (TBN), Spm, RIF, INH, BDQ, and para-aminosalicylic acid (PAS) were purchased from Sigma-Aldrich. Stocks of a concentration ranging from 10 to 100 mM (depending on the saturation point) were made in DMSO (or methanol for PAS) and stored at −20°C. During specific experiments, these were further diluted in nuclease-free water to achieve the desired concentrations.

### Drug testing

The minimum inhibitory concentrations (MICs) of the tested antibiotics and the fractional inhibitory concentrations (FICs) of their combination were determined using a resazurin-based checkerboard assay as previously described (30, 31). The following criteria were used to conclude the nature of their interactions. Synergism (FIC ≤ 0.5), additive (0.5 < FIC ≤ 1), indifference (1 < FIC < 4), and antagonism (FIC ≥4) (30). All 96-well plates used during drug testing were incubated in a 37°C/5% CO_2_/95% humidity incubator to allow optimal bacterial growth without evaporation, which could have compromised the interpretation of the results.

### Survival assay

The results from the drug testing described above guided the determination of optimal concentrations to be used for validation by the CFU-based method. Logarithmic-phase cultures were diluted to about 10^4^–10^5^ CFUs/mL mycobacteria, which were treated with defined concentrations of antibiotics: 90 µM Spm, 52 nM RIF, Spm-RIF, and untreated/no antibiotics; 90 µM Spm, 90 nM INH, Spm-INH, and untreated/no antibiotics; 90 µM Spm, 38 nM PAS, Spm-PAS, and untreated/no antibiotics; or 90 µM Spm, 390 nM BDQ, Spm-BDQ, and untreated/no antibiotics. Treated and untreated samples were exposed for 7 days and CFUs count were determined post-exposure. To investigate the effect of Spm on the combination of RIF and INH, bacteria were treated with either Spm or INH, RIF or IHN-RIF, RIF-Spm, INH-Spm, RIF-INH-Spm, or no antibiotics (untreated control). Three methods were used in this study to investigate the combined effect of all three antibiotics. This was achieved either by measuring the fluorescence intensity of resazurin (Ex-540/35, Em-590/20 nM) added to the wild-type H37Rv as previously described (32) or by measuring the fluorescence intensity (Ex-575/15, Em-635/32) of the mCherry-expressing H37Rv. The method relying on the fluorescence of resazurin was performed over 7 days at the following concentrations: 90 µM Spm, 92 nM INH, and 26 nM RIF, and the results from this method were validated by the CFU-based method. For the method relying on the fluorescence of mCherry, the following concentrations were used: 16 µM Spm, 46 nM INH, and 13 nM RIF, and exposure was for over 13 days, and the results from this method were further validated by the CFU-based method as described above. To understand the effect of oxidative stress (OS) or nitrosative stress (NS) on the activity of Spm, a concentration of 2 mM Spm and/or 2 mM CuOOH (OS) (33) and/or 10 mM TBN (NS) (34) was used to expose bacteria prepared as described above for 3 hours. An interaction was considered additive if the percentage survival was halved when compounds were combined and synergistic when the percentage survival dropped to less than half the percentage survival of the corresponding single treatment controls.

### Reactive oxygen species quantification

To measure the level of reactive oxygen species (ROS) resulting from the combined effect of CuOOH and Spm, logarithmic-phase cultures diluted to an OD_600_ of 0.2–0.3 (10^7^–10^8^ CFUs/mL) were treated with either 2 mM Spm alone or CuOOH alone or Spm-CuOOH, including the corresponding DMSO control of each condition. The bacterial suspensions were treated with a final concentration of 10 µM H2DCF, mixed by a vortex, and aliquoted in dark (optical-bottom) 96-well plates, in three to six replicates. The fluorescence intensity of the product generated by H2DCF during oxidative stress was measured every 10–30 minutes (Ex-485/20, Em-528/20 nM) over a period of 4 hours by a plate reader at 37°C as previously described (31). To analyze the data obtained, the H2DCF-ROS fluorescence intensity of each condition was normalized to its respective control. Similarly, when the effect of pH on the ability of Spm to generate ROS was investigated, the pH of Sauton’s media was adjusted to a range of pH, and the results were normalized to their respective untreated pH controls since changes in pH itself seemed to influence the fluorescence intensity of H2DCF. Likewise, in order to investigate the effect of iron concentrations on the ability of Spm to generate ROS, phosphate-buffered saline was spiked with a range of concentrations of ferric ammonium citrate (NH_4_)_5_[Fe(C_6_H_4_O_7_)_2_], (FeAmCi), and measurements of ROS were made relative to the untreated control of each iron concentration. To investigate the combined oxidative stress effect of Spm with known antibiotics, the level of ROS was measured over 3–4 hours on logarithmic cultures diluted to an OD_600_ of 0.2–0.3 and treated with a final concentration of 100–250 µM of Spm and/or each antibiotic. When lower concentrations of antibiotics were used, as low as half the MICs, a lower number of bacteria (10^5^ CFUs/well of bacteria, about 100× less) were tested over a longer (7 days) period. An exact replicate of each condition was stained in parallel with resazurin. The end-point fluorescence of H2DCF and resazurin was measured 24–48 hours post-staining. To analyze the data obtained, the relative fluorescence unit (RFU) of H2DCF for each condition was normalized to the corresponding resazurin RFU. An interaction was considered additive if the ROS level of the combination was the sum of the ROS levels of both tested compounds and synergistic if it was more than the sum of both.

### NADP/NADH and iron measurements

The levels of NADP and NADPH produced during Spm stress were measured using the NADP/NADPH fluorometric assay kit (Abcam) according to the manufacturer’s instructions, with slight modifications as follows. Bacteria cultured to the logarithmic phase were diluted to an OD_600_ of 0.2. After centrifugation, the collected bacterial pellet was resuspended in an equal volume of the lysis buffer. These were incubated for at least 1 hour on a heating block [37°C, with agitation (200 rpm)]. Cell lysates were collected by centrifugation, and supernatants were stored at −20°C or used directly for NADP/NAPH level quantification according to the manufacturer’s instructions.

For the quantification of the levels of ferric (Fe^3+^) iron and ferrous (Fe^2+^) iron produced during Spm stress, the culture OD_600_ was between 0.8 and 1 since lower optical densities were below the detection limit. A volume of 100 µL of either the Spm-treated sample or the control was aliquoted in a 96-well plate. Then, ferric iron and ferrous iron were quantified using an iron assay kit (Sigma-Aldrich Co. LLC) following the manufacturer’s instructions.

### Reverse transcriptase PCR

A volume of 10 mL of early to mid-logarithmic-phase cultures was treated either with 80 µM Spm or with no antibiotic (untreated control) and harvested 3 hours later (after incubation at 37°C) using the RNA Pro Blue kit (MP Bio). RNA was extracted and purified, and reverse transcriptase PCR (RT-PCR) was performed as previously described (manuscript under revision). The forward primer, reverse primer, and probe used for the RT-PCR amplification and quantification of *rv0327c* [cytochrome P450 135A1 (Cyp135A1)] were CGGAGCATGGGCAAGTT, CCTCCCGCCGTATCGA, and FAM-CACGCCTACTAAGCCCTCCAG-BBQ, respectively, and those of *rv1394c* [cytochrome P450 132 (Cyp132)] were CCAGATCCAGCGTGTCGTAAA, GGACTCGTCGGTCTGATGAT, and FAM-TCGCAAGATCGTGCATGGACTG-BBQ, and those of the housekeeping gene sig A (*rv2703*) were CAAACAGATCGGCAAGGTAG, CTCGATCCGCTTGGCTAG, and FAM-CTCAACGCCGAGGAAGAGGTC-BBQ.

### Statistical analysis

Statistical analyses were performed with Prism (version 10.0) using either one-way ANOVA (Bonferroni’s post-test) or two-way ANOVA (Bonferroni’s post-test) or a *t*-test approach (unpaired), with alpha set to 0.05 (*P < 0.05, **P < 0.01, ***P < 0.001, and ****P < 0.0001).

## RESULTS AND DISCUSSION

### Spermine induces the formation of reactive oxygen species

**Fig 1:**
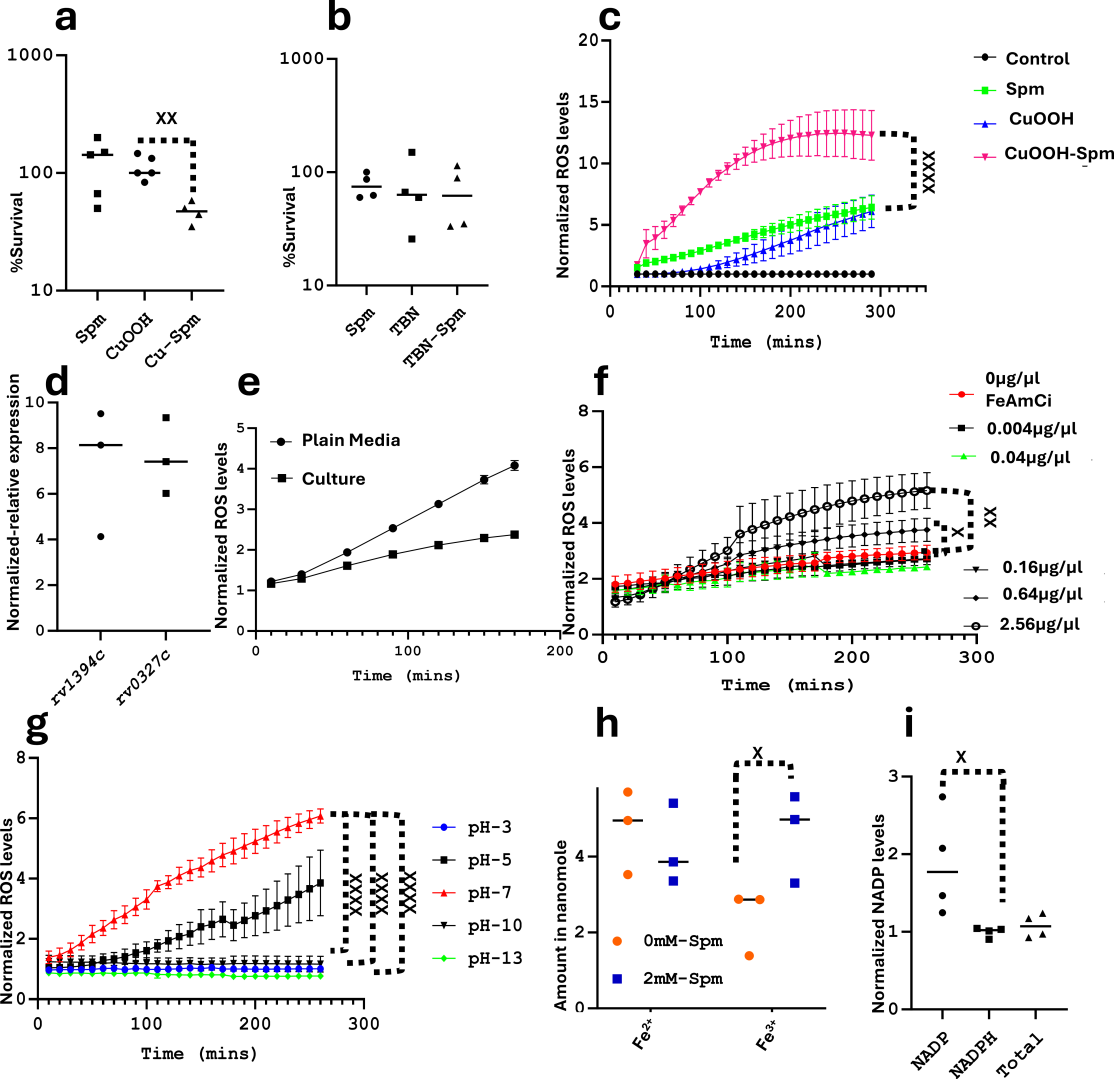
Physiological and biochemical effects of toxic amounts of spermine. (a) Spermine enhances the activity of CuOOH. Bacteria were exposed to either Spm or CuOOH or both. The percentage survival was derived from the CFU count of each condition relative to their respective controls. The results are representative of four to five biological replicates (BRs). An unpaired multiple *t*-test was performed on Prism 10, leading to *P* = 0.002. (b) Spermine does not enhance the activity of TBN. Experiments were performed in the same way. The results are representative of four biological replicates. (c) Spermine enhances the level of ROS generated by CuOOH. Experiments were performed as described in panel a, and the level of ROS was measured over time. The results are representative of two biological replicates and 13 technical replicates (TRs). Two-way ANOVA was used to derive a *P* value of less than 0.0001 for most time points. (d) Spermine induces the overexpression of *rv0327c* (cytochrome P450 135A1) and *rv1394c* (cytochrome P450 132). Cultures were treated with either 2 mM Spm or the exact percentage of DMSO (untreated control). The expression of these genes was estimated in each condition and normalized to the expression level of the housekeeping gene *sigA*. Then, the relative expression of each gene in the treated condition was normalized to the expression level of the corresponding gene in the untreated condition. The results are representative of three BRs. (e) Spermine generates ROS in both bacteria-free media and cultures. Cultures or plain media were treated in duplicate with or without Spm (DMSO control). The production of ROS increased both in the culture and in the media but to a lesser extent in the culture. (f) High concentrations of iron enhance ROS generation by Spm. Ferric ammonium citrate (FeAmCi) was added at different concentrations to PBS, and the level of ROS generated by Spm over time was measured. The results are representative of three BRs and seven TRs. Two-way ANOVA (mixed effect) was performed to derive a *P* value of 0.03 when PBS at 0.64 µg/µL FeAmCi was compared to PBS with no iron and to derive a *P* value of 0.0024 when PBS at 2.56 µg/µL FeAmCi was compared to PBS with no iron. (g) The activity of Spm is optimal at pH 7. Sauton’s media were prepared at different pH values, and the level of ROS generated by Spm was measured over time in the various media. The results are representative of three BRs and six TRs. Two-way ANOVA (mixed effect) was performed to compare the ROS level at pH 7 to other pH values (*P* < 0.0001). (h) Spm induces a high production of ferric iron. Different species of iron were measured in Spm-treated and untreated M.tb cultures, and the relative amount in nanomole was calculated based on a standard curve derived from a standard provided in the kit used. The results are representative of three BRs. Two-way ANOVA with a Bonferroni two-stage linear post-test was performed to derive a *P* value of 0.03. (h) Spm induces a higher production of NADP. In this case, the levels of either NADP or NADP or the total levels in the treated samples were normalized to their levels in the untreated samples. The results are representative of four BRs. Two-way ANOVA with a Bonferroni post-test was performed to derive a *P* value of 0.036.

**Fig 2:**
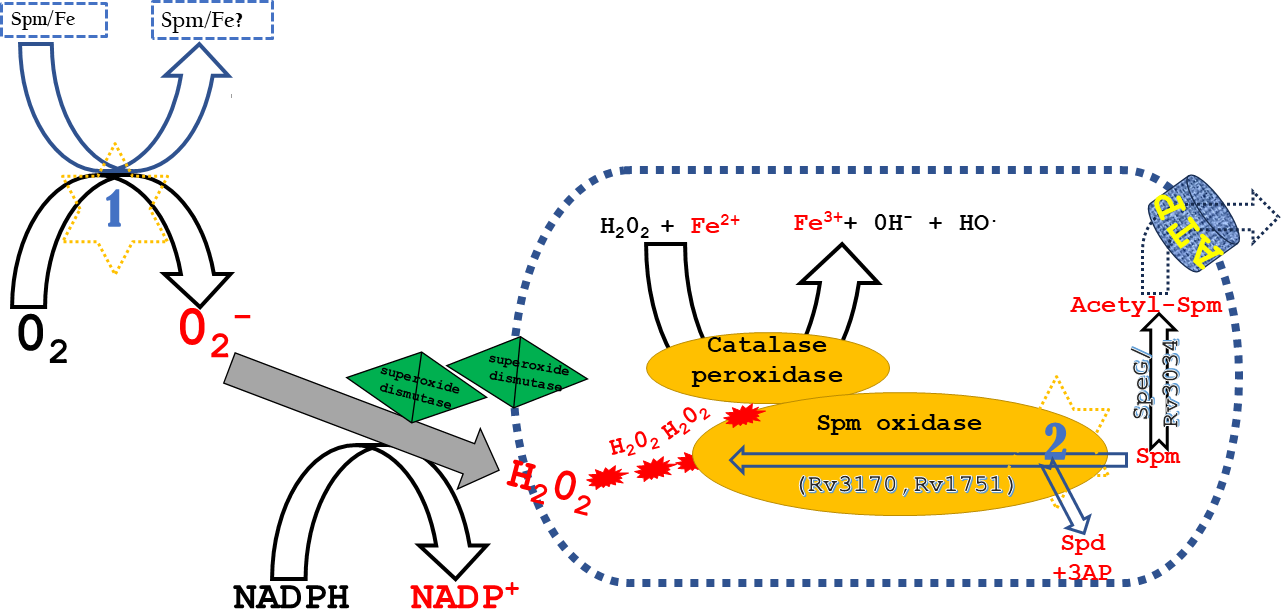
Suggested mechanism of ROS generation by spermine. Spermine (Spm) can spontaneously generate superoxide in the presence of iron and oxygen; however, it remains unclear how (reaction 1). The generated superoxide is converted to hydrogen peroxide by superoxide dismutase produced by M.tb. Hydrogen peroxide is therefore converted by catalase-peroxidase to hydroxyl radicals at the expense of ferrous iron, generating ferric iron. However, Spm can also produce hydrogen peroxide by the enzymatic reaction of spermine oxidase (SPO) (reaction 2), yielding spermidine (Spd) and 3-acetamidopropanal (3AP). The M.tb proteins that share the highest similarities to the eukaryotic SPO are Rv3170 and Rv1751. M.tb can also detoxify Spm through acetylation by SpeG (ortholog is Rv3034). The acetylated product is therefore excreted by ATP-dependent transporters.

During phagocytosis of M.tb, an oxidative burst occurs where reactive oxygen species and reactive nitrogen species (ROS and RNS) are generated by macrophages against M.tb (35). We therefore sought to know if Spm is able to increase the susceptibility of M.tb to ROS or RNS. To achieve this, we exposed M.tb to either Spm and/or an ROS donor (CuOOH) and/or an RNS donor (TBN). Spermine was able to significantly enhance the sensitivity of M.tb to CuOOH (Fig. 1a) but did not enhance the sensitivity of M.tb to the RNS donor (Fig. 1b). Furthermore, there was no statistically significant difference between the treatment with Spm alone and the combined Spm-CuOOH treatment, either due to the experimental outlier (Table S1; Fig. 1a) or it is possible that CuOOH is able to only marginally enhance the sensitivity of M.tb to Spm. Nevertheless, the general trend indicates that combining Spm with CuOOH enhances their activity (Table S1). In order to further understand how Spm enhanced the antimycobacterial activity of the ROS donor CuOOH, we measured the level of ROS generated by M.tb under the tested conditions. While Spm and the ROS donor individually induced ROS formation, their combination resulted in an additive enhancement of ROS production (Fig. 1c). Intrigued by this observation, we sought to know if this was reflected in the expression of genes encoding enzymes involved in the redox homeostasis of M.tb. In view of previous studies, which indicated that cytochrome P450 activity induces oxidative stress (36, 37), and also in line with previous studies showing the upregulation of cytochrome P450-related genes during oxidative stress and/or antibiotic stress in M.tb (36, 38), we selected cytochrome P450 135A1 (*rv0327c*) and cytochrome P450 132 (*rv1394c*) to study their expression levels during Spm stress (by RT-PCR). We found that they were upregulated (Fig. 1d). It remains unclear if these genes were upregulated because Spm directly targets the cytochrome P450 complex of M.tb or if it is because of an alteration in energy levels during Spm stress. However, in view of previous studies indicating that increased activity of the cytochrome P450 complex leads to increased oxidative stress (36, 37), we had at least genetic information (Fig. 1d) that supported the generation of oxidative stress by Spm that we observed biochemically (Fig. 1c). Furthermore, we found in our previous studies (manuscript under revision) that the expression of a few genes encoding enzymes involved in the redox homeostasis of M.tb was altered during Spm stress in media different from the media we used in this study (7H9 versus Sauton’s media). The genes encoding AhpC (39) and AhpD (39) were downregulated, while the gene encoding WhiB7 (40) was upregulated (manuscript under revision). It remains unclear if the expression of genes encoding all or most enzymes involved in the redox homeostasis of M.tb is altered during Spm stress in the media tested in this study (Sauton’s media). However, as previously shown, there is not always a direct correlation between the expression of genes encoding redox homeostasis-related enzymes and oxidative stress (41, 42). For example, *mshA*, a gene essential for the biosynthesis of mycothiol (required for the detoxification of ROS) (43), is not upregulated during oxidative stress in M.tb (41, 42); however, the production of mycothiol is elevated during oxidative stress and during treatment by an ROS-inducing antibiotic (44, 45). Therefore, more studies are required to clearly elucidate the transcriptomic, proteomic, and metabolic changes caused by Spm stress, which is beyond the scope of this study. However, we were able to show that at toxic concentrations, Spm is able to induce ROS formation and is able to enhance the activity of ROS donors against M.tb (Fig. 1). It was previously indicated that Spm can act as an anti-oxidant, protecting DNA from free radicals (46). However, this was not shown in cells but on molecules of DNA at sub-lethal, non-toxic concentrations. Therefore, the tendency of Spm to generate ROS is more likely a mode of toxicity to cells when administered at lethal concentrations. This theory was confirmed in recent studies (47), demonstrating that at low concentrations, Spd (a precursor of Spm) is able to act as an anti-oxidant (protecting cells and promoting growth) but becomes a pro-oxidant at toxic concentrations in *Escherichia coli* (47). To further understand the mechanism of ROS production by Spm, we first investigated if Spm was able to spontaneously generate ROS in bacteria-free (plain) media, and we found that Spm was able to spontaneously generate ROS in plain media (Fig. 1e) and to a lesser extent in bacterial culture (Fig. 1e), probably due to the tightly regulated anti-oxidants produced by M.tb (48, 49). Similarly, previous studies have indicated the ability of Spd to spontaneously generate ROS in media containing iron and oxygen (47); however, it still remains unclear how. To gain further insights into ROS generation by Spm, we investigated if that was dependent upon the media/environment composition, such as iron concentration and pH. We found that at concentrations higher than the standard iron concentration in media (0.04 µg/µL), the level of ROS was significantly elevated (Fig. 1f). Next, we found that the production of ROS by Spm was optimal at pH 7 and decreased at lower and higher pH values (Fig. 1g). To get further insights into the mechanism of ROS production by Spm, we measured the levels of ferric iron, ferrous iron, NADP, and NADPH. We found that Spm was able to significantly increase the levels of ferric iron (Fig. 1h) and NADP (Fig. 1i). In light of these findings and previous studies (47, 50), we could envisage the following putative mechanism of ROS generation by Spm (Fig. 2). At physiological pH and optimal oxygen and iron levels, Spm spontaneously generates ROS (more likely superoxide according to previous studies on Spd) (47). The generated superoxide is converted to hydrogen peroxide by superoxide dismutase (produced by M.tb), leading to an increased production of NADP (Fig. 1i; Fig. 2). The generated hydrogen peroxide is in turn converted by catalase-peroxidase to hydroxyl radicals at the expense of ferrous iron, causing an increase in ferric iron (Fig. 1h; Fig. 2). On the other hand, as previously shown (50), Spm can also generate ROS enzymatically when spermine oxidase (SPO) converts Spm to Spd, leading to the production of hydrogen peroxide and 3-acetamidopropanal (Fig. 2). The produced hydrogen peroxide is again converted to hydroxyl radicals by catalase-peroxidase (Fig. 2). Spermine oxidase has never been characterized in M.tb. However, we searched and found Rv3170 (putative flavin-containing monoamine oxidase AofH) and Rv1751 (probable oxidoreductase) as the closest orthologs to the eukaryotic SPO when blasted against the M.tb proteome. Acetylation of Spm by SpeG and excretion of the acetylated product are another route used by *Escherichia coli* (47, 51 – 53) to detoxify Spm and Spd. We found that the closest ortholog of *E. coli* SpeG in M.tb is Rv3034c (Fig. 2), an M.tb acetyltransferase that was found to regulate host oxidative stress levels by inducing peroxisome homeostasis (54). Excretion and transport of Spd and Spm require energy/ATP-dependent (active) transporters in both bacteria (55) and eukaryotes (3), which was also revealed in our previous studies (manuscript under revision) by the upregulation of genes encoding ABC (ATP-dependent) transporters during Spm stress in M.tb (manuscript under revision). A versatile biological role of Spm has been reported (3) (discussed in the introduction). Therefore, it is safe to state that since Spm is able to alter a wide range of biological processes (3), it is also able to use various means to exert its antimycobacterial activity and enhance the activity of other antimycobacterial agents. In this study, we were able to investigate only one (ROS generation) of the many modes of action of Spm. Therefore, the investigation of other modes of action of Spm in future studies is in order.

### Spermine enhances the activity of rifampicin

**Fig 3:**
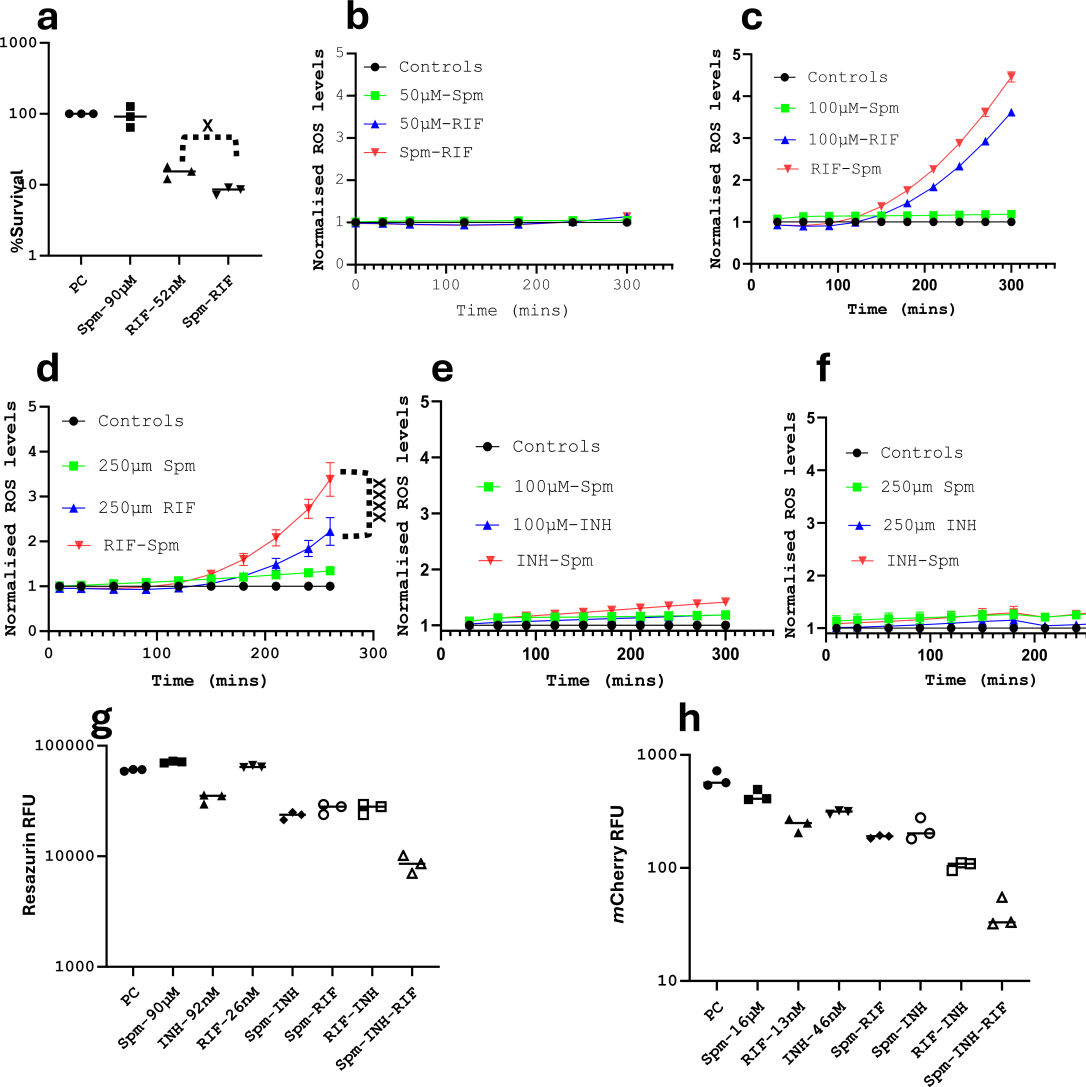
The effect of the combination of Spm, RIF, and/or INH. (a) Spm enhances the activity of RIF. Bacteria were exposed to either Spm and/or RIF and plated 7 days later for the CFUs count. The percentage survival was derived by dividing the CFUs (multiplied by 100) of each condition to the untreated positive control (PC). An unpaired *t*-test was performed to derive a *P* value of 0.018 between RIF alone and RIF-Spm. Data are representative of two technical replicates (TRs) and two biological replicates (BRs). (b) ROS generated by 50 µM RIF are undetectable within 4 hours. Bacteria were treated with 50 µM of each antibiotic, and the ROS released over time were measured and normalized to the ROS released in the controls. The results are representative of four TRs. (c) ROS generated by 100 µM RIF are detectable within 4 hours. Bacteria were treated with 100 µM of each antibiotic, and the ROS released over time were measured and normalized to the ROS released in the controls. Spm was able to marginally enhance the ROS produced by RIF at this concentration. Data are representative of two TRs. (d) Spm enhances ROS production by RIF. Bacteria were treated in this case with 250 µM of each antibiotic. A two-way ANOVA statistical test was performed to derive a *P* value of ≤0.0001. Data are from two BRs and nine TRs. (e) INH does not induce a significant amount of ROS. Experiments were performed, and the results were derived as described in panel c. (f) The combination of Spm and INH does not induce a significant production of ROS at higher concentrations. Experiments were performed, and the results were derived as described in panel d. The results are representative of two BRs and seven TRs. (g) Evaluation of the combination of RIF-INH-Spm by resazurin fluorescence. Bacteria were treated with either one antibiotic or two or three antibiotics. Resazurin was added to the different conditions 7 days later, and the fluorescence of resazurin was measured 24–48 hours later. Data are representative of three TRs. Statistical analyses were not performed because data were derived only from one BR; however, the same conditions were validated by the CFU count (Fig. S4, left panel). (h) Evaluation of the combination of RIF-INH-Spm by mCherry fluorescence. Bacteria expressing mCherry were treated with lower concentrations of antibiotics for 13 days instead of 7 days. The fluorescence of viable cells was measured. Data are representative of three TRs. Statistical analyses were not performed because data were derived only from one BR; however, the same conditions were validated by the CFU count (Fig. S4, right panel).

Rifampicin is an antibiotic that targets the β-subunit of RNA polymerase (56), and it is one of the first-line drugs used to treat TB (8). We therefore sought to know if Spm could enhance the activity of RIF. We initially performed a checkerboard assay, which revealed that Spm was able to reduce the MIC of RIF and vice versa (Table 1; Fig. S1), resulting in an additive effect. Validations by the CFU-based method (Fig. 3a) revealed that the percentage survival of bacteria treated with RIF at the tested concentration was 15.2 ± 2.4%, while it was 8.2 ± 0.8% when added to Spm, further confirming the additive effect (Fig. 1a; Table 1). The biological relevance of this additive activity remains to be shown with more in-depth studies, such as *in vivo, ex vivo,* and omics investigations. Nevertheless, since it was previously indicated that additive activities can be useful in some treatments (57, 58), we sought to understand the mechanism of their additive effect. Previous studies have demonstrated the ability of RIF to generate ROS in M.tb. However, ROS measurements were performed 24–72 hours post-exposure to 1–10 µM RIF (~10–100× its MIC) using electron paramagnetic resonance (EPR) spin trapping (59), a method that is more sensitive and more stable than the dye-based methods (59). Therefore, using the H2DCF dye-based method, we initially wanted to see if the same result could be reproduced after a shorter exposure (3–5 hours instead of 72 hours) to RIF. Therefore, we tested a range of concentrations of RIF and/or Spm and/or INH to evaluate the detection limit of ROS formation using our method (Fig. S2; Fig. 3b). We could only detect increased ROS formation by RIF at a concentration of 100 µM (Fig. 3c) under the tested conditions. The high detection limit of our method is due to the low sensitivity of the dye-based ROS detection method (versus the EPR method) (59, 60) and is probably because measurements were performed within a shorter exposure (4 hours instead of 72 hours). After optimizing our method to obtain results that align with previous studies, we sought to know if Spm enhanced the production of ROS by RIF (59), possibly explaining their additive antimycobacterial activity (Table 1; Fig. 3a). We noticed that at 100 µM, Spm slightly enhanced the production of ROS by RIF, but Spm did not visibly induce ROS production on its own at that concentration. We repeated the experiment at 250 µM and found that Spm was able to significantly enhance the ROS generated by RIF at this concentration (Fig. 3d) but was still able to only marginally generate ROS on its own at this concentration. The difficulty in seeing the obvious ROS production by Spm alone at 250 µM is more likely due to its high MIC and the low sensitivity of our method. Our dose-response curves in our previous study (manuscript under revision) and our current study (Table 1) indicate that Spm is bacteriostatic in Sauton’s media, with MICs ranging from 100 to 500 µM. This is probably because of the composition of the culture media since previous studies reported a bactericidal effect instead (23). Therefore, the effect of Spm is more likely dependent on the cell density. Since we used a higher (100×) cell density for the measurement of ROS (compared to the density used for the determination of MIC; please see Materials and Methods), it would be justifiable that a slightly higher concentration would be required to elicit a visible effect. Moreover, if the ROS generated by RIF alone was only detected at a concentration of about 1,000× its MIC using our method (Fig. 3c) and 10–100× its MIC by the previously shown more sensitive method (59), then to be able to detect ROS generated by Spm at a concentration that is close to its MIC (Table 1) only proves that the ability to generate ROS by Spm is its primary mechanism of action. It is not a result of a secondary effect through the interference of a particular biological process of mycobacteria, as is the case with RIF, which generates ROS as a secondary effect resulting from its binding to the β-subunit of RNA polymerase (56). This is supported by our finding that Spm is able to spontaneously generate ROS in the tested media, without any direct contact with the bacteria (Fig. 1e). In brief, using our method, we confirmed that RIF was able to induce the formation of ROS, as previously shown (38, 59, 61, 62), and found that this was enhanced by Spm (Fig. 3d). This is in accord with previous studies demonstrating that RIF is able to synergize with CuOOH in terms of antimycobacterial activity and ROS production (61), while it is able to increase the expression of some cytochrome P450 genes (38), as seen with Spm in this study (Fig. 1d). Therefore, the additive activity of Spm and RIF found in this study (Table 1; Fig. 2) is at least partially due to the ability of Spm to enhance ROS generated by RIF. However, as discussed above, due to the versatility of Spm, we cannot exclude other possible mechanisms responsible for their additive activity.

**TABLE 1:** Checkerboard results of the combination of Spm with drugs

	BR-1^*a*^	BR-2	BR-3
**Spm and RIF**			
Spm-MIC (µM)	208	208	104
RIF-MIC (nM)	13	26	80
Spm-RIF-MIC	13–6.5	26–6.5	26–40
∑FIC	0.6	0.6	0.75
Conclusion	Additive	Additive	Additive
**Spm and INH**			
Spm-MIC (µM)	208	208	104
INH-MIC (nM)	42	42	140
Spm-INH-MIC	104–10	104–10	52–70
∑FIC	0.73	0.73	1
Conclusion	Additive	Additive	Additive
**Spm and BDQ**			
Spm-MIC (µM)	260	521	260
BDQ-MIC (µM)	0.375	0.375	0.375
Spm-BDQ-MIC	33–0.09	130–0.09	130–0.09
∑FIC	0.366	0.366	0.56
Conclusion	Synergistic	Synergistic	Additive
**Spm and CCC**			
Spm-MIC (µM)	260	208	521
CCC-MIC (nM)	1,000	1,000	830
Spm-CCC-MIC	33–500	104–500	130–420
∑FIC	0.63	1	0.75
Conclusion	Additive	Additive	Additive
**Spm and PAS**			
Spm-MIC (µM)	260	260	130
PAS-MIC (nM)	62.5	38	19
Spm-PAS-MIC	65–7.8	33–9	33–5
∑FIC	0.366	0.364	0.5
Conclusion	Synergistic	Synergistic	Synergistic

^*a*^ BR, biological replicate (experiment performed on different days).

**Fig 4:**
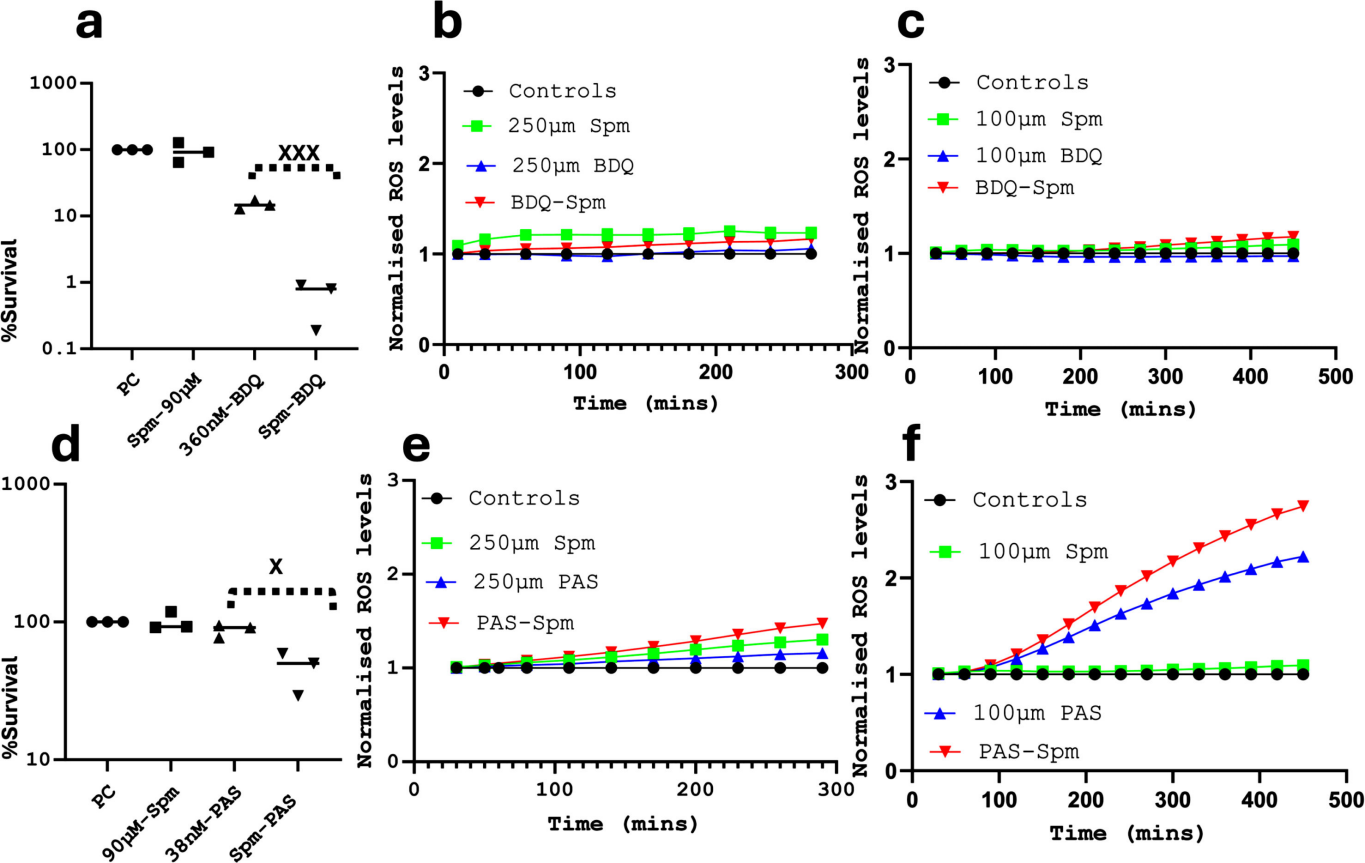
The effect of the combination of Spm and BDQ or PAS. (a) Spm significantly enhances the activity of BDQ. Bacteria were exposed for 7 days at the defined concentrations of antibiotics. Viability was estimated by CFUs count, and the percentage survival was derived by dividing the CFUs count of each condition (multiplied by 100) to the control. Data are representative of three TRs and two BRs. An unpaired *t*-test was performed to compare the BDQ-alone treatment to the Spm-BDQ treatment, giving a *P* value of 0.0004. (b) BDQ does not enhance the production of ROS by Spm. Samples were treated with 250 µM of each (or both) antibiotic(s), and the level of ROS was measured over 3–4 hours. BDQ seemed to decrease the ROS levels instead. Data are representative of five TRs. (c) BDQ does not enhance the production of ROS by Spm even after prolonged exposure. Bacteria were treated with lower concentrations (100 µM), and the ROS produced were measured over a longer period. However, BDQ does not seem to increase the production of ROS by Spm. Data are representative of four TRs. (d) Spm significantly enhances the activity of PAS. Experiments were performed, and data were analyzed as described in paned a. Data are representative of three TRs and two BRs. An unpaired *t*-test was performed to compare the BDQ-alone treatment to the Spm-BDQ treatment, giving a *P* value of 0.0162. (e) PAS marginally enhances the production of ROS by Spm during short exposure. Samples were treated, and data were analyzed as described in panel b. Data are representative of four TRs (f) PAS enhances the production of ROS by Spm after prolonged exposure. Samples were treated, and data were analyzed as described in panel c. Data are representative of four TRs.

### Spermine enhances the combined activity of isoniazid and rifampicin

Isoniazid is another first-line drug used to treat M.tb infections (8). It targets InhA, which is involved in the elongation of fatty acids during cell wall biosynthesis (63). We therefore also sought to know if Spm could enhance the activity of INH. The checkerboard drug testing revealed that INH was able to reduce the MIC of Spm and vice versa but by only 50% as opposed to the combination of RIF and Spm that decreased their MICs to less than 50% (Table 1; Fig. S3a). Further validations by the CFU-based method also revealed just a marginal decrease in CFUs when INH and Spm were combined (Fig. S3b). The measurement of ROS during INH treatment revealed that INH does not induce a significant amount of ROS, and combining it with Spm does not enhance the production of ROS (Fig. 3e), even when used at high concentrations (Fig. 3f). This is in accord with previous studies, showing that the activity of INH is linked with the induction of RNS (nitrosyl radicals) (64) but not ROS, as is the case with RIF (59). Spm did not enhance the activity of the tested RNS donor (Fig. 1b), which could explain its inability to enhance the activity of INH. However, since RIF and INH are both first-line drugs used to treat TB, we sought to know if adding Spm to a combination of INH and RIF would enhance their combined activity. We investigated their combined effect through measurements of the fluorescence intensity of resazurin (Fig. 3g) of the stained wild-type M.tb and the fluorescence intensity of the mCherry-expressing M.tb strain (Fig. 3h). Both testing methods revealed that combining all three (RIF, INH, and Spm) enhanced their activities (Fig. 3g and 2h). This was further validated by the CFU-based method (Fig. S4). It was previously shown that RIF is able to induce lipid peroxidation (altering the membrane integrity (61) of mycobacteria) and that it is able to synergize with CuOOH, which is an organic ROS donor affecting mainly lipids (33). Therefore, Spm, which is also able to enhance the activity of CuOOH (Fig. 1a), may induce lipid peroxidation as well. Therefore, it is possible that RIF and Spm are able to cause lipid peroxidation in M.tb, thereby damaging its cell envelope (65). Isoniazid, on the other hand, does not alter the cell membrane by lipid peroxidation but by targeting InhA, which is involved in the elongation of fatty acids of the cell wall of M.tb. Therefore, the enhanced activity of the combination of RIF, INH, and Spm could at least be partially associated with their combined effect on the membrane of M.tb. The enhanced activity of the combination of RIF, INH, and Spm could also be associated with the enhanced activity and/or production of catalase-peroxidase. The ROS generated by Spm and RIF would require catalase-peroxidase during detoxification, while INH is activated by KatG (64) (encoding catalase-peroxidase). Therefore, the more active or the higher the production of KatG, the more likely INH is to be activated. However, these possible mechanisms remain speculative until they are demonstrated in future studies.

### Spermine enhances the activity of bedaquiline

Because resistance to BDQ has already been reported (10), we sought to know if Spm could enhance the activity of BDQ. Our checkerboard analyses revealed that Spm significantly decreased the MIC of BDQ and vice versa (Table 1; Fig. S5), resulting in a synergistic interaction, a result that was validated by the CFU-based method (Fig. 4a), as the percentage survival resulting from the treatment with BDQ at the tested concentration was 14.9 ± 1.8% but became 0.6 ± 0.3% when Spm was added to the treatment (Fig. 3a). However, measurements of ROS production during Spm and/or BDQ treatments revealed that their synergistic activity is not primarily associated with increased ROS production (Fig. 3b and c). Therefore, their synergy is probably due to other physiological effects. Bedaquiline inhibits ATP synthesis at the last node of the electron transport chain (ETC) of M.tb (66). On the other hand, there are numerous studies supporting the ATP-dependent transport of Spm (3, 55). Moreover, in our previous studies (manuscript under revision), we identified the upregulation of genes encoding ABC transporters (ATP-dependent) during Spm stress. To investigate if it was related to the ability of Spm to require ATP-dependent transport, since BDQ inhibits ATP synthesis (66), we investigated the combined activities of Spm and carbonyl cyanide m-chlorophenylhydrazone (CCC), a proton motive force inhibitor, which has been used intensively in ATP-dependent drug efflux studies in M.tb (67). It was able to reduce the MIC of Spm and vice versa (Table 1; Fig. S6). Therefore, if the detoxification of Spm by efflux is ATP-dependent as previously indicated (3, 55) (manuscript under revision), then it is reasonable that an ATP synthesis inhibitor such as BDQ would enhance the activity of Spm and vice versa. Furthermore, polyamines have been shown to bind to ATP, and Spm is the polyamine with the strongest ATP-binding affinity (68, 69), supporting further the role of Spm in the regulation of energy metabolism. Taken together, these findings suggest that the synergistic activity of the combination of Spm and BDQ could be linked to their ability to modulate ATP levels in M.tb. This could explain why the interaction of Spm with BDQ has a greater effect on M.tb (Table 1; Fig. 3 and 4) as compared to other tested antibiotics in this study (Table 1; Fig. 3 and 4). It is more likely that, in this case, it is not only Spm that enhances the activity of BDQ by altering ATP levels but also BDQ that enhances the activity of Spm by inhibiting the synthesis of ATP, which is required for the detoxification of Spm through active export by ABC pumps. RIF for instance may not interfere with the detoxification of Spm but exerts its additive effect by an additional generation of ROS (besides the ROS generated by Spm), which are usually detoxified by the tightly regulated anti-oxidative system of M.tb, further pointing to the versatility of Spm in its mode of action, which is not limited to ROS generation. Therefore, more studies are required to decipher other mechanisms of action (and drug synergism) of Spm.

### Spermine enhances the activity of para-aminosalicylic acid

Para-aminosalicylic acid is an antibiotic recommended (by the WHO) for the treatment of extensively drug-resistant TB (XDR TB) (8). Since the treatment of XDR TB is difficult, we sought to know if Spm could enhance the activity of PAS. Our checkerboard analyses revealed that Spm significantly decreased the MIC of PAS and vice versa, resulting in a synergistic interaction (Table 1; Fig. S7). This was further validated by the CFU-based method (Fig. 4d), where the percentage survival of bacteria treated with PAS was 87.4 ± 7.5% and the percentage of bacteria treated with PAS and Spm was 46 ± 12.5%, revealing an additive interaction in this case. Although the conclusive interaction is slightly different, both methods show that Spm enhances the activity of PAS. To investigate if this effect is at least partially due to increased levels of ROS in the bacteria, we measured the level of ROS generated during the treatment of M.tb by either PAS and/or Spm. We found that PAS was able to only marginally increase the level of ROS generated by Spm over 3 hours (Fig. 4e), as opposed to RIF (Fig. 3c). However, when we exposed the bacteria to a lower concentration of PAS over a longer period, the generation of ROS by PAS was observed (Fig. 4f; Fig. S8), concurring with previous studies showing that the inhibition of folate biosynthesis by PAS or sulfonamides leads to increased cellular ROS (45, 70, 71). In addition, ROS production by PAS was enhanced in the presence of Spm (Fig. 4; Fig. S8). As opposed to RIF and Spm, where the generation of ROS was an immediate reaction happening within 3 hours (Fig. 3c), the generation of ROS by folate inhibitors only occurs after prolonged exposure (45) (Fig. 4e and f; Fig. S8). This is likely because ROS generation in this case is not an immediate consequence of the mode of action of PAS but a tertiary downstream effect caused by the inhibition of folate biosynthesis. Folate is involved in the metabolism of homocysteine, which is in turn required for the biosynthesis of low molecular weight thiols, which are required to maintain the cellular redox state (72). Therefore, the synergistic activity of the combination of Spm and PAS could at least be partially due to their ability to both induce the production of cellular ROS through different mechanisms. Nevertheless, we cannot exclude the possibility of additional (or other) mechanisms of synergism.

### Conclusion

In summary, we have shown for the first time that Spm is able to generate ROS in M.tb and attempted to elucidate its mechanisms of ROS generation. Although we have not completely deciphered the full picture of the mechanism of the antimycobacterial activity of Spm, we have demonstrated for the first time that it is able to enhance the activity of some anti-tuberculosis drugs. This study paves the way for future thorough investigations on the suitability and efficacy of Spm as a potential adjunct TB therapy.

## Data Availability

A Prism file containing all data and their analyses described in this manuscript can be accessed at Figshare using DOI: 10.6084/m9.figshare.24659331

## AFTER EPUB

*[This article was published on 14 December 2023 with an incomplete reference. The References were corrected in the current version, posted on 20 December 2023.]*

## SUPPLEMENTAL MATERIAL

Supplemental material spectrum.03568-23-s0001.pdf Table S1 and Fig. S1 to S8.

ASM does not own the copyrights to Supplemental Material that may be linked to, or accessed through, an article. The authors have granted ASM a non-exclusive, world-wide license to publish the Supplemental Material files. Please contact the corresponding author directly for reuse.
