# Supplementary material for: Spermine enhances the activity of anti-tuberculosis drugs
Source: Microbiol Spectr. 2023 Dec 14;12(1):e03568-23. doi: 10.1128/spectrum.03568-23 (PMC10782994; doi:10.1128/spectrum.03568-23)
Supplement: Supplemental material — Table S1 and Fig. S1 to S8. [file spectrum.03568-23-s0001.pdf]

Spermine is a pro-oxidant that enhances the activity of anti-tuberculosis drugs

Carine Sao Emani[1], Norbert Reiling[1,2]
[1]Microbial Interface Biology, Research Center Borstel, Leibniz Lung Center, Parkallee 1-40, Borstel, Germany.
[2]German Center for Infection Research (DZIF), Partner Site Hamburg-Lübeck-Borstel-Riems, Borstel, Germany
Contact:
carine.emani.sao@gmail.com, nreiling@fz-borstel.de

Supplementary Table.1

Table.1 Percentage (%) survival of M.tb under various treatments

	**CuOOH**	**Spm**	**CuOOH-Spm**
Experiment-1 (BR-1)	133.33	66.67	35
Experiment-2 (BR-2)	100	50*	50
Experiment-3 (BR-3)	100	200	44.44
Experiment-4 (BR-4)	83.33	150	Data lost
Experiment-5 (BR-5)	146.7	142.86	58.33
Average ± Standard deviation	112.67 ± 23.52	121.90 ± 55.76	46.94 8.48

*An outlier caused possibly a technical artefact

Supplementary Figure.1

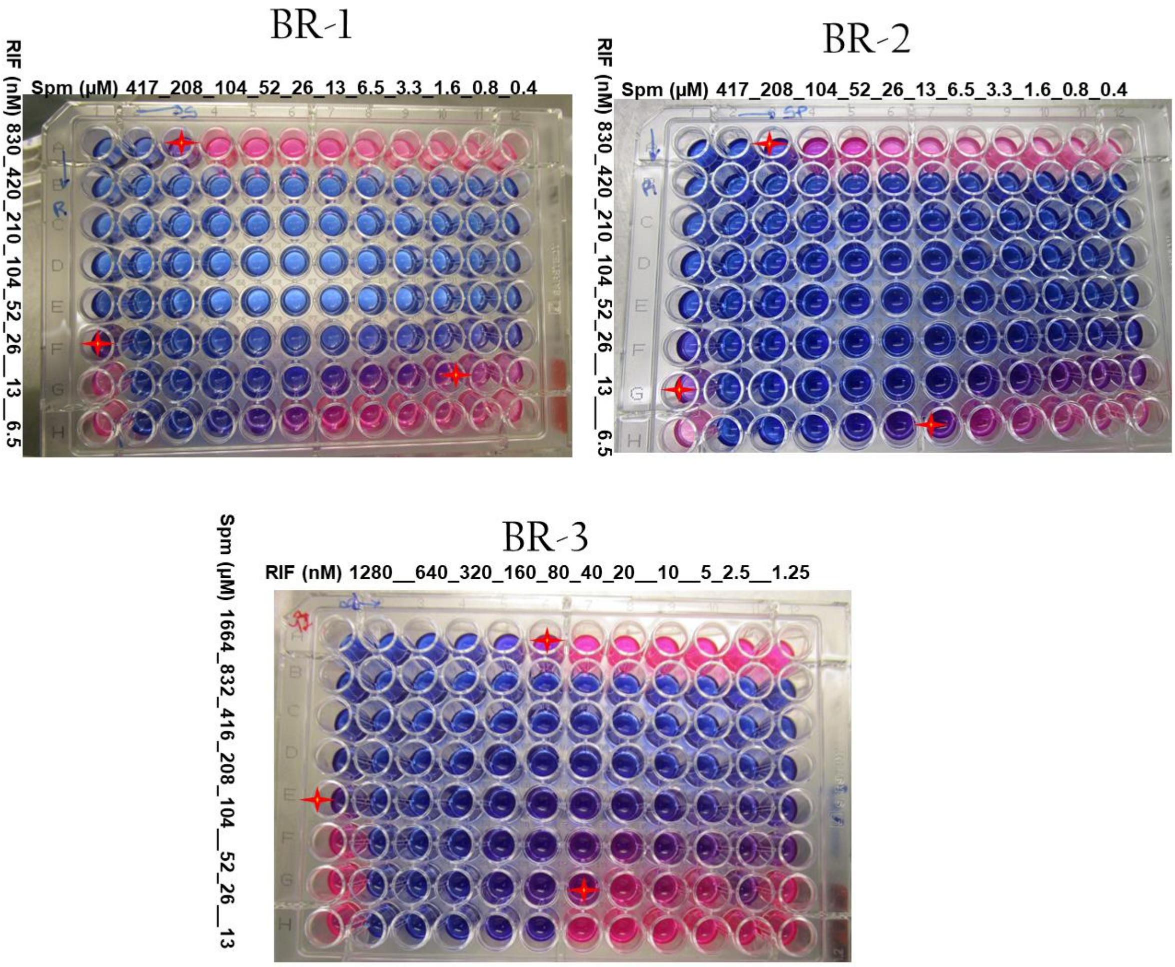

Supplementary Figure 1. Checkerboard assay showing the additive activities of RIF and Spm. The first column starting from cell A1 displays the MIC (indicated by the star) of RIF only (BR1 and BR2), or Spm only (BR3), while the first row, starting from cell A2 displays the MIC of Spm only (BR1 and BR2) or RIF only (BR3). The MIC of both antibiotics when combined is indicated by the star in other areas of the plate. **BR:** biological replicate, experiment performed on different days

Supplementary Figure.2

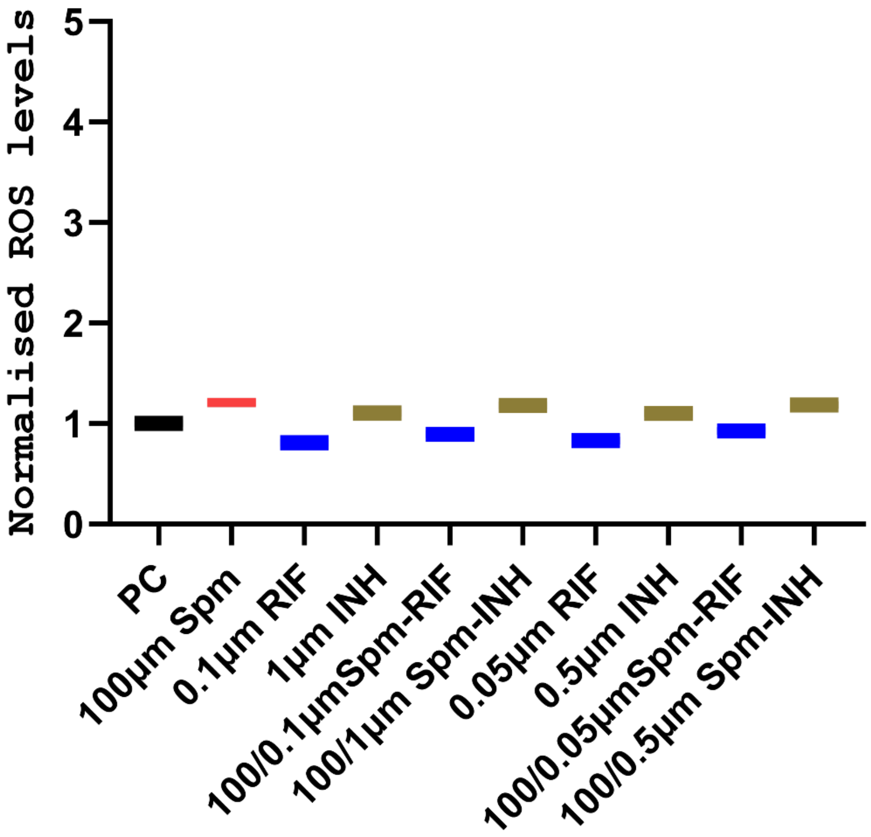

Supplementary Figure 2. ROS levels measured after exposure of M.tb to antibiotics for approximately 4 hours using the dye (H2DCF)-based method. Bacteria were treated with 100µM Spm and/or 0.1µM RIF, 1µM INH, 0.05 µM RIF, 0.5 µM INH, and lower concentrations not shown here. ROS levels were below detection limit with the tested concentrations.

Supplementary Figure.3

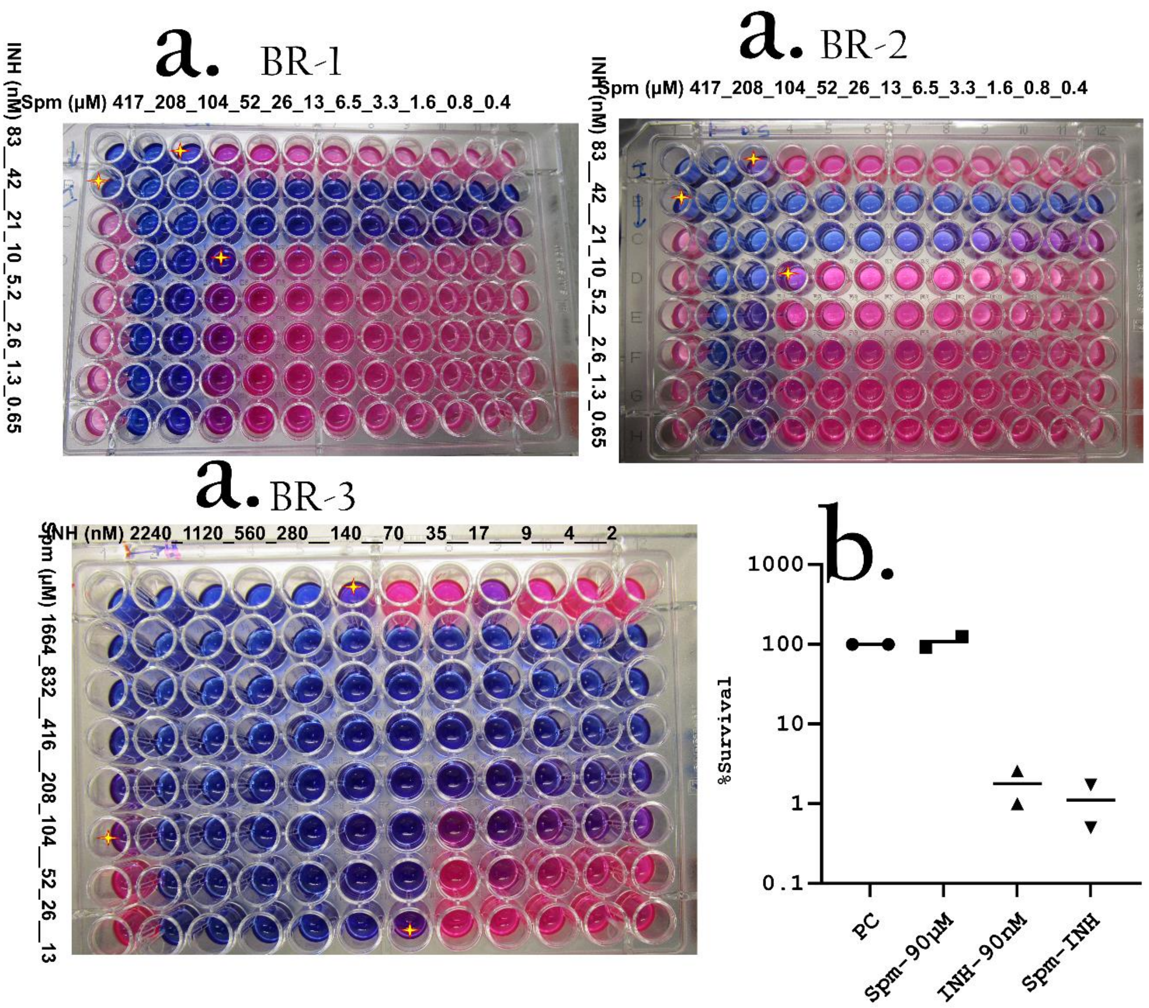

Supplementary Figure 3. Interaction of Spm with INH. 3a. Checkerboard assay showing the additive activities of INH and Spm. The first column starting from cell A1 displays the MIC (indicated by the star) of INH only (BR1 and BR2), or Spm only (BR3), while the first row, starting from cell A2 displays the MIC of Spm only (BR1 and BR2) or INH only (BR3). The MIC of both antibiotics when combined is indicated by the star in other areas of the plate. **3b. Validation by the CFUs based method**. Bacteria were exposed to either Spm and/or INH, and plated 7 days later for CFUs count. The percentage survival was derived by dividing the CFUs (multiplied by 100) of each condition to the untreated positive control (PC). Data are representative of 2 independent experiments (2 BRs).

Supplementary Figure.4

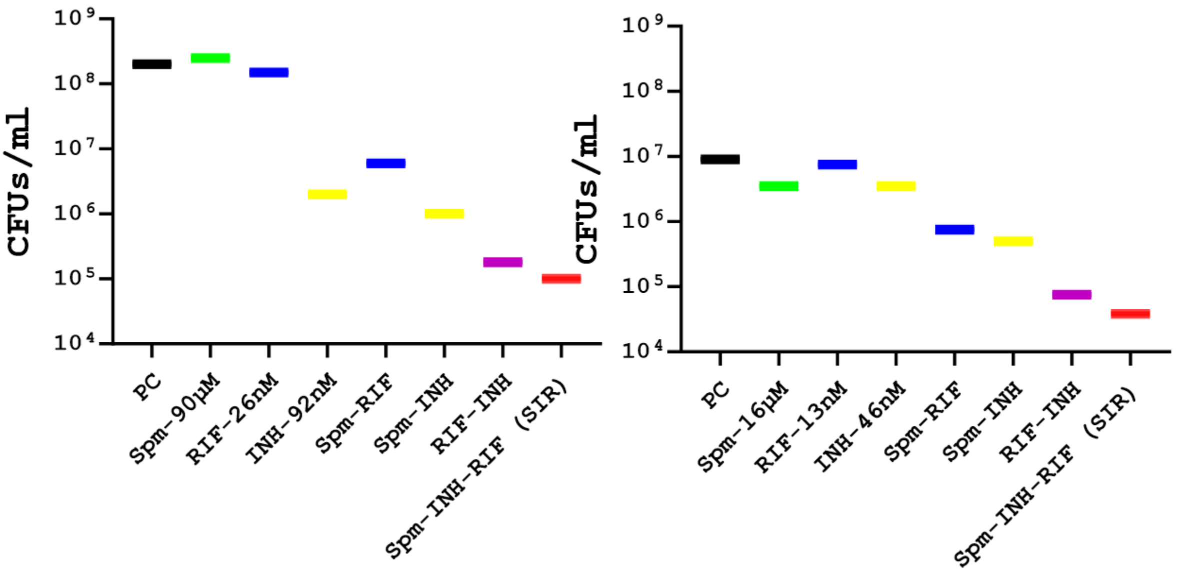

Supplementary Figure.4 Enhanced activity of the combination of RIF-INH-Spm demonstrated by CFUs count. In the left panel, bacteria were exposed to indicated concentrations for 7 days and plated for CFUs estimation per condition. In the right panel, bacteria were exposed at lower concentrations for 14 days and results remain the same.

Supplementary Figure.5

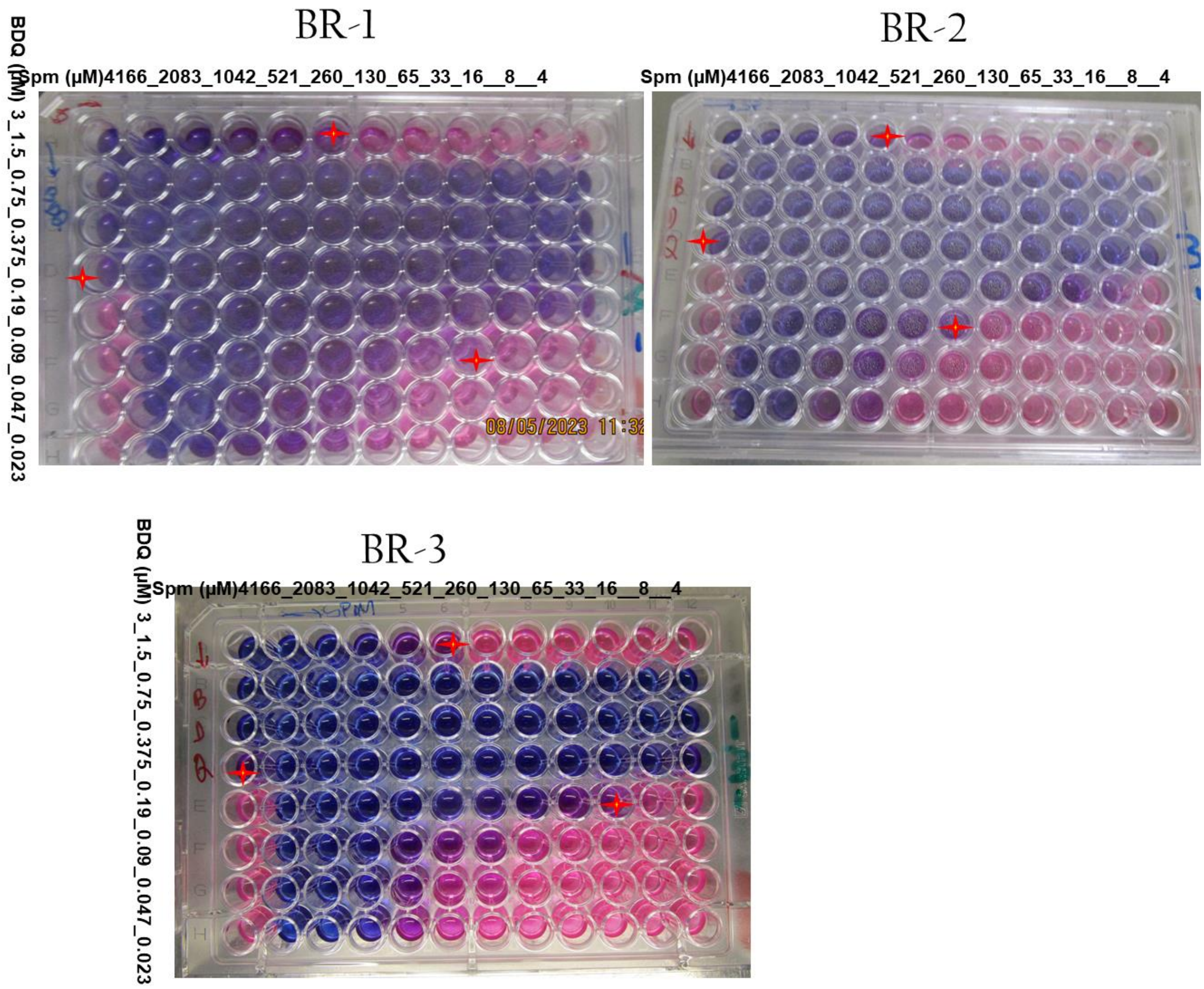

Supplementary Figure 5. Checkerboard assay showing the synergistic activities of BDQ and Spm. The first column starting from cell A1 displays the MIC (indicated by the star) of BDQ only, while the first row, starting from cell A2 displays the MIC of Spm only. The MIC of both antibiotics when combined is indicated by the star in other areas of the plate.

Supplementary Figure.6

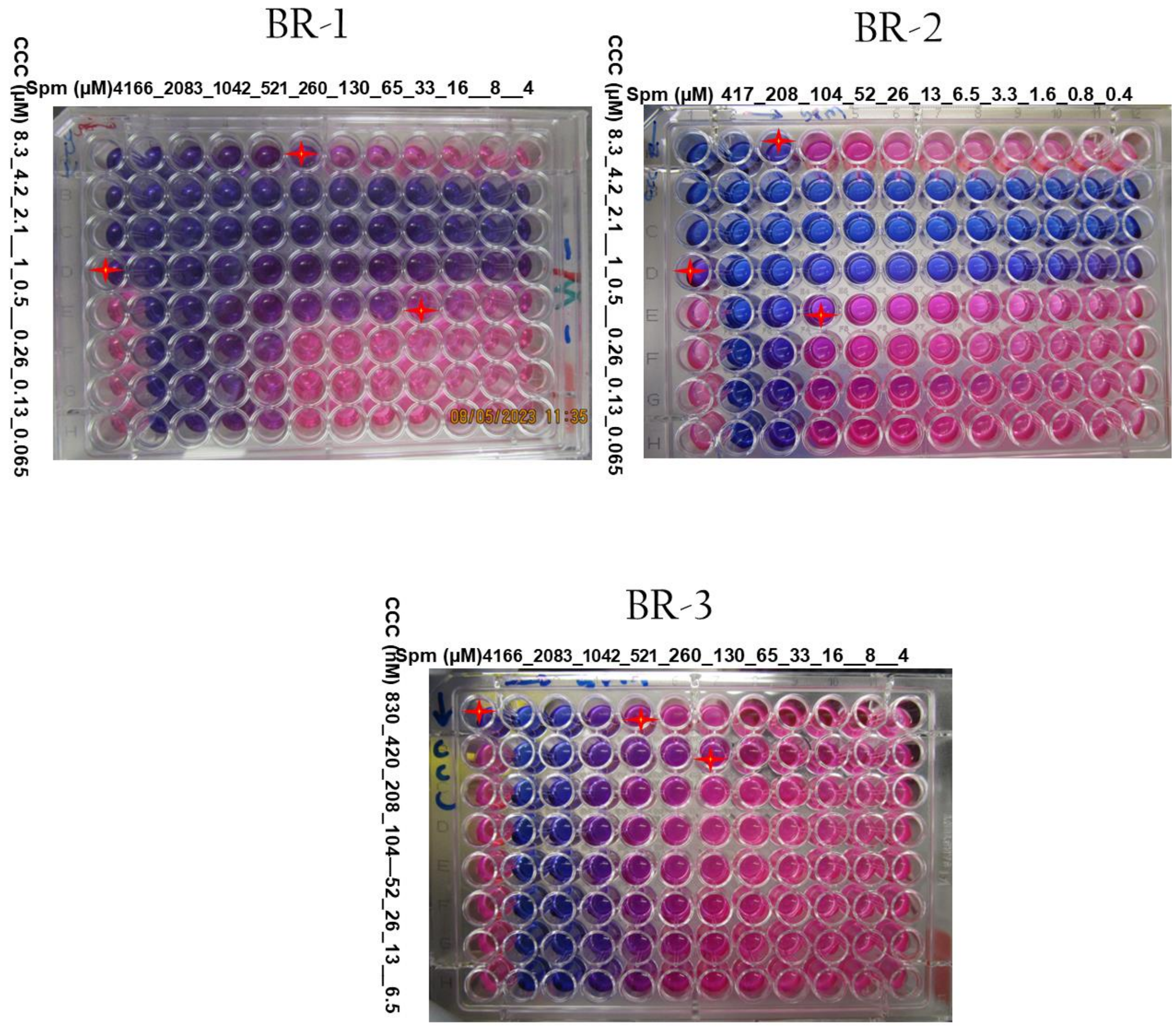

Supplementary Figure 6. Checkerboard assay showing the additive activities of CCC and Spm. The first column starting from cell A1 displays the MIC (indicated by the star) of CCC only, while the first row, starting from cell A2 displays the MIC of Spm only. The MIC of both antibiotics when combined is indicated by the star in other areas of the plate.

Supplementary Figure.7

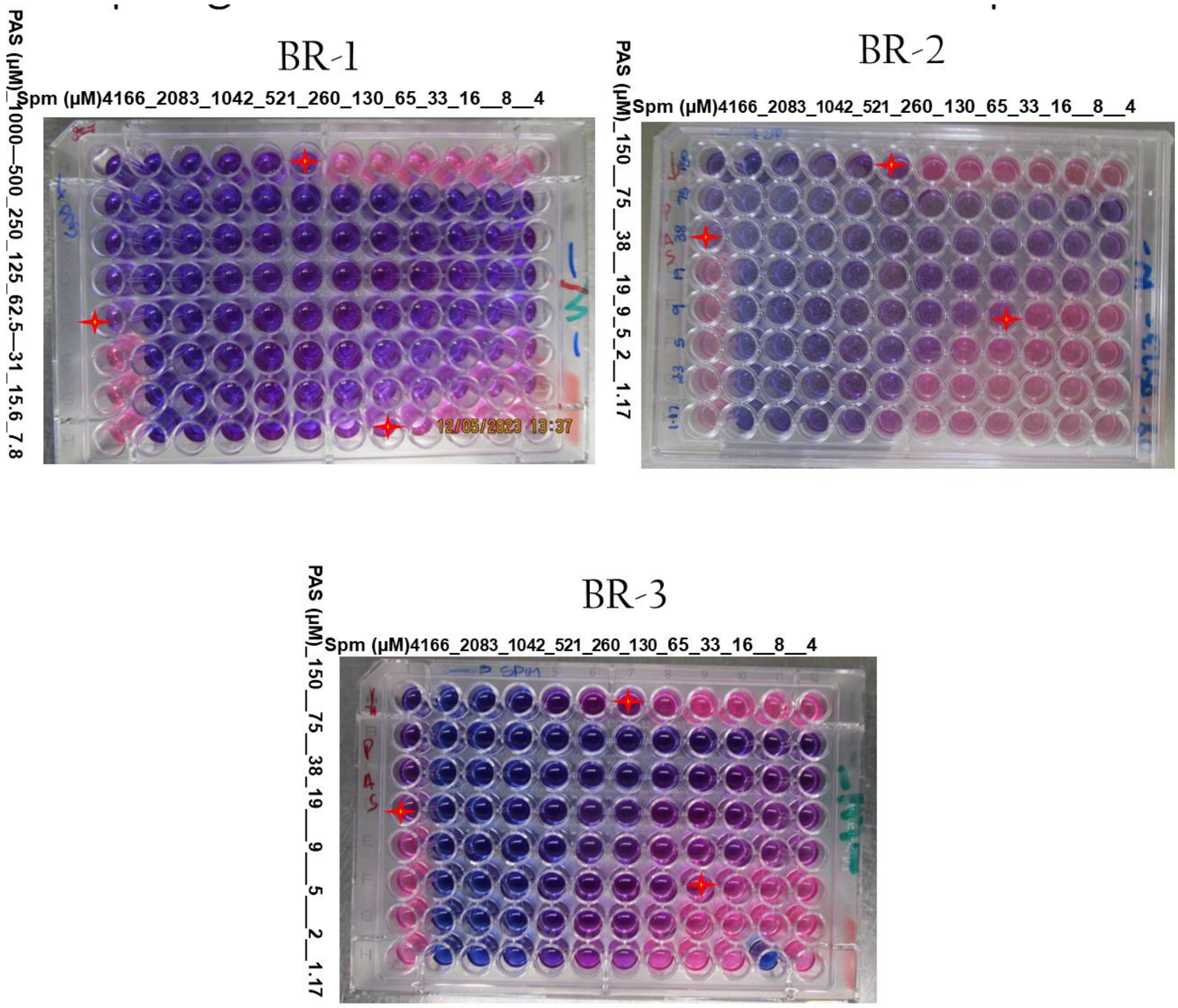

Supplementary Figure.7 Checkerboard assay showing the synergistic activities of PAS and Spm. The first column starting from cell A1 displays the MIC (indicated by the star) of BDQ only, while the first row, starting from cell A2 displays the MIC of Spm only. The MIC of both antibiotics when combined is indicated by the star in other areas of the plate.

Supplementary Figure.8

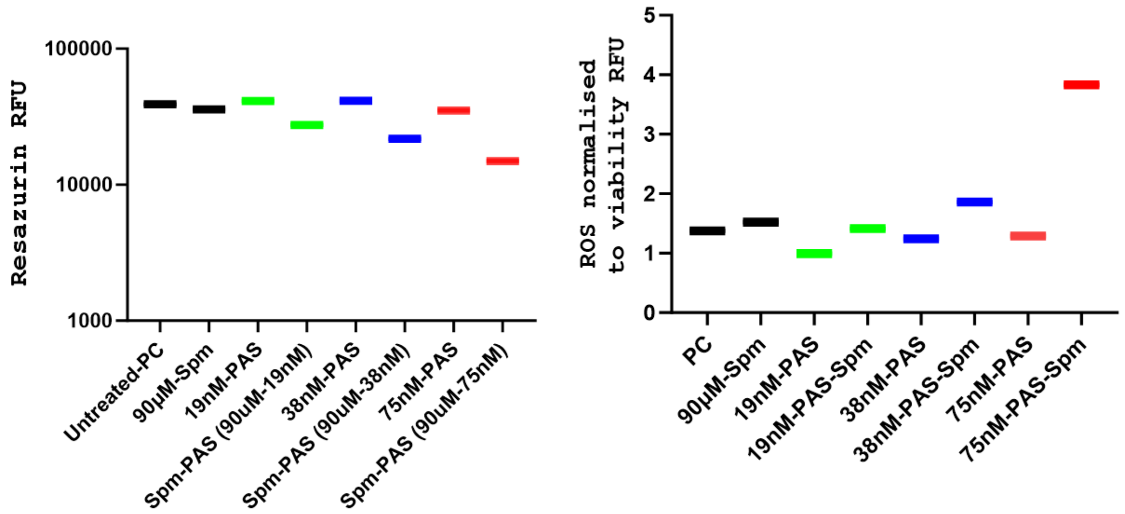

Supplementary Figure.8 Spermine enhances the activity of PAS and the production of ROS by PAS. On the left panel, a range of concentrations within the MIC of PAS was tested with and without Spm over 7-9 days. There was an enhanced activity of PAS when combined with Spm, irrespective of the concentration. Viabilities were estimated by the relative fluorescence unit (RFU) of resazurin. On the right panel, another replica of the same sample was stained with H2DCF and the RFU of H2DCF was normalized to the corresponding resazurin RFU.
